# Rational allocation of resources available for healthcare: understanding cost effectiveness analyst

**DOI:** 10.7448/IAS.17.4.19492

**Published:** 2014-11-02

**Authors:** Andrew Briggs

**Affiliations:** Health Economics & Health Technology Assessment, Institute of Health & Wellbeing, University of Glasgow, Glasgow, UK

## Abstract

Health systems around the world are grappling with the difficult issue of how to allocate scare healthcare resources. Within Europe many different approaches to healthcare resource allocation exist, but it is clear that the formal techniques of cost-effectiveness analysis are becoming increasingly important. This presentation will first address the fundamental economic principles of scarcity and opportunity cost, before looking at how different European systems are attempting to address the economic challenge of providing healthcare for their population from a limited budget. The presentation will go on to consider general issues in health economic evaluation that are likely important for clinical community focused on HIV treatment: appropriate comparator treatments for defining opportunity cost, patient heterogeneity and the potential for personalized/stratified medicine to control costs and target treatment appropriately.

